# Assessment of Metabolic, Inflammatory, and Immunological Disorders Using a New Panel of Plasma Parameters in People Living with HIV Undergoing Antiretroviral Therapy—A Retrospective Study

**DOI:** 10.3390/jcm13154580

**Published:** 2024-08-05

**Authors:** Beata Szymańska, Brygida Knysz, Hubert Ciepłucha, Agnieszka Piwowar

**Affiliations:** 1 Department of Toxicology, Faculty of Pharmacy, Wroclaw Medical University, 50-556 Wroclaw, Poland; agnieszka.piwowar@umw.edu.pl; 2 Department of Infectious Diseases, Liver Diseases and Acquired Immune Deficiencies, Faculty of Medicine, Wroclaw Medical University, 51-149 Wroclaw, Poland; brygida.knysz@umw.edu.pl (B.K.); hubert.cieplucha@umw.edu.pl (H.C.)

**Keywords:** HIV, combined antiretroviral therapy, carbohydrate metabolism, lipid metabolism, inflammation

## Abstract

**Background/Objectives:** People living with HIV (PLWH) treated with combined antiretroviral therapy (cART) show a greater predisposition to metabolic and inflammatory disturbances compared to the general population. This study aimed to assess the effect of five years of cART use on the level of selected parameters related to carbohydrate and lipid metabolism and inflammation in PLWH compared to the uninfected. **Methods:** The levels of sirtuins (-1, -3, -6); irisin (IRS); myostatin (MSTN); peptide YY (PYY); glucagon-like peptide-1 (GLP-1); dipeptidyl peptidase IV (DPP-4); fetuin-A (FETU-A); pentraxin 3 (PTX3); chemokine stromal cell-derived factor 1 (SDF-1); regulated on activation, normal T cell expressed and presumably secreted (RANTES); and interleukins (-4, -7, -15) in the plasma of PLWH and a control group were evaluated by immunoassay methods. The results obtained after five years of antiretroviral therapy were compared with the levels obtained before and one year after cART. **Results:** Analysis of the parameters after five years of cART showed significantly higher levels in PLWH compared to the control group for SIRT-6, IRS, and IL-4 and significantly lower levels for RANTES and IL-7. There were significantly higher levels of SIRT-6, PYY, GLP-1, and PTX3 obtained after five years of cART compared to the results before therapy and after one year of cART. **Conclusions:** The results indicated changes occur in the expression of selected parameters during cART use in PLWH. Further research on the clinical usefulness of selected parameters and obtaining new information on the development of HIV-related comorbidities needs to be conducted.

## 1. Introduction

The introduction of combined antiretroviral therapy (cART), involving the use of a combination of two or three antiretroviral drugs, has led to a radical improvement in the health of people living with HIV (PLWH) and preventing disease progression in people without clear clinical symptoms of infection [1]. The implementation of cART has contributed to a significant improvement in the life expectancy of people infected with HIV. However, the need for the long-term use of cART is associated with an increased development of metabolic disorders and a higher risk of developing type 2 diabetes, insulin resistance, osteoporosis, kidney disease, and cardiovascular disease [2].

The occurrence of metabolic diseases is a serious problem in people living with HIV [3,4]. Metabolic complications associated with antiretroviral therapy, including dyslipidemia, insulin resistance, and altered fat tissue distribution (lipodystrophy), are well-documented side effects of drugs such as PIs and some NNRTIs. Infected people not using cART have a decrease in total cholesterol, LDL, and HDL without an effect on TG. When cART was used, an increase in total cholesterol and LDL was observed; the effects of treatment on TG depend on the therapeutic regimen used. These effects on the lipid profile have been demonstrated with the use of NRTIs. The use of TAF (tenofovir alafenamide) and TDF (tenofovir disoproxil) have been shown to significantly contribute to lipid metabolism disorders, with TAF-based regimens showing a more atherogenic lipid profile [3].

Diabetes is a common disease among PLWH. Studies conducted in the United States have estimated diabetes to occur in 19.3% of infected women and 12.2% of infected men using cART therapy for more than 6 months. Although the incidence of diabetes is not significantly higher compared to the general population, the treatment regimen may affect carbohydrate metabolism and the occurrence of T2DM, while the impact of the infection itself on carbohydrate metabolism remains unknown [4].

The presence of HIV promotes chronic activation of the immune system and inflammation. This results from several factors, including thymic dysfunction, persistent antigenic stimulation due to low residual viremia, microbial translocation, dysbiosis due to intestinal mucosal disruption, co-infections, and the toxicity of cART. All these factors lead to uncontrolled immune system activation and inflammation, which may lead to an increased risk of comorbidities not related to AIDS [5].

Despite the high prevalence of these conditions, limited research is available assessing such disorders in PLWH taking cART. This inspired us to conduct this retrospective study. The paper presents 15 parameters that are not routinely measured and are related to the regulation of carbohydrates, lipid metabolism, inflammation, and the response of the immune system. The investigated parameters related to metabolic disorders included sirtuin 1 (SIRT-1), sirtuin 3 (SIRT-3), sirtuin 6 (SIRT-6), irisin (IRS), myostatin (MSTN), peptide YY (PYY), glucagon-like peptide 1 (GLP-1), and dipeptidyl peptidase 4 (DPP-4). Parameters associated with inflammation and regulation of the immune system included fetuin-A (FETU-A), pentraxin 3 (PTX3), stromal descent factor 1 (SDF-1), RANTES chemokine, interleukin 4 (IL-4), interleukin 7 (IL-7), and interleukin 15 (IL-15).

The sirtuin family includes seven proteins (SIRT-1–SIRT-7) that participate in metabolic processes through the post-translational modification of proteins [6]. Sirtuins influence numerous cellular processes such as DNA repair, carcinogenesis, inflammation, apoptosis, energy metabolism (glucose and lipid metabolism, in which SIRT-1 and SIRT-3 play a key role), and oxidative stress (mainly SIRT-1, SIRT-3, and SIRT-6) [7,8].

IRS is an adipomyokine and is important in regulating metabolism and energy expenditure [9]. IRS has anti-inflammatory properties and can increase insulin sensitivity while inhibiting gluconeogenesis [10].

MSTN is a factor that inhibits myoblast proliferation, which determines the final number and size of muscle fibers [11]. Increased MSTN concentrations predispose to the development of metabolic disorders such as insulin resistance [12].

PYY is a polypeptide that regulates the gut–brain axis, inhibiting intestinal motility, gastric emptying, secretion of gastric juices, and appetite. An inverse correlation has been demonstrated between PYY levels and the development of cardiovascular risk factors (diabetes, hypertension, and hypercholesterolemia) and the risk of cardiovascular events [13,14].

GLP-1 is an incretin peptide with hypoglycemic effects. It works by increasing the release of insulin from the pancreas and inhibiting the release of glucagon [15].

DPP-4 is an exopeptidase involved in the regulation of blood glucose levels by hydrolyzing two incretin peptide hormones, GLP-1 and gastric inhibitory polypeptide (GIP) [16]. The HIV Tat protein has a significant affinity for DDP-4, weakening its enzymatic activity, and has immunosuppressive properties [17].

FETU-A is a negative acute-phase protein observed to decrease during inflammation. FETU-A is an inhibitor of insulin receptor tyrosine kinases in muscle, liver, and adipose tissues, which promotes the development of insulin resistance and type 2 diabetes mellitus (T2DM) [18].

PTX3 is a multifunctional acute-phase protein and a key factor regulating the innate immune response [19].

SDF-1 plays a key role in regulating the inflammatory response, myelopoiesis, and HIV pathogenesis [20]. SDF-1 is considered a prognostic factor for the insufficient restructuring of the immune system despite the use of effective antiretroviral treatment [21].

Regulated on activation, normal T cell expressed and presumably secreted (RANTES, also known as CCL5 and CC motif chemokine ligand 5) is a chemokine synthesized by T lymphocytes that is a mediator of the inflammatory process and has chemotactic properties against immune system cells [22].

IL-4 is a type II inflammatory cytokine; it acts antagonistically on interferon-gamma (IFNγ) and also inhibits the release of pro-inflammatory cytokines [23].

IL-7 is a cytokine whose main function is to regulate the development of immune cells, including B lymphocytes, T lymphocytes, and natural killer cells [24].

IL-15 plays a key role in antiviral defenses and against non-viral intracellular pathogens [25].

This study aimed to assess the impact of cART on changes in the concentrations of 15 selected parameters (SIRT-1,-3, and -6; IRS; MSTN; PYY; GLP-1; DPP-4; FETU-A; PTX3; SDF-1; RANTES; IL-4; IL-7; and IL-15) related to the regulation of carbohydrate and lipid metabolism, the development of inflammation, and the immune system response in PLWH after 5 years of antiretroviral therapy compared to people without HIV. Since the study is a continuation of previously performed measurements, this study aimed to compare the current results with those obtained before the implementation of cART and one year after the use of antiretroviral treatment. This study also took into account the impact of the cART regimen used, looking specifically at the effects of integrase strand transfer inhibitors (INSTIs) vs protease inhibitors (PIs) after 5 years of use on tested metabolic parameters and immune status, as well as the age of PLWH.

## 2. Material and Methods

### 2.1. Study Group Characteristics

The study group consisted of men living with HIV (MLWH) and men without HIV of similar age (from 28 to 58 years), constituting the control group, from the Lower Silesia Region of Poland.

Inclusion criteria for the MLWH group included a confirmed HIV infection and antiretroviral medication use for 5 years. The exclusion criteria for the MLWH group were the presence of chronic diseases such as diabetes, cardiovascular diseases, urinary system diseases, cancer, and the use of drugs other than antiretroviral drugs.

The inclusion criteria for the control group included a confirmed absence of HIV infection, no active viral or bacterial infections, and no chronic diseases such as diabetes, cancer, cardiovascular diseases, or urinary tract diseases, as well as no constant use of medications. The ages of participants were selected from a pool of similarly aged men.

Co-infection with hepatitis B and C viruses, chronic infections, and sexually transmitted infections were not detected in the MLWH and control groups.

HIV-infected men were treated with two therapeutic regimens, which included two nucleoside reverse transcriptase inhibitors and NRTIs (emtricitabine and tenofovir alafenamide) in combination with a PI (ritonavir-boosted lopinavir or cobicistat-boosted darunavir) or INSTI (dolutegravir).

Data on HIV RNA viral loads, CD4+T lymphocyte and CD8+T lymphocyte counts, CD4+/CD8+ ratios, the therapeutic regimen, and biochemical parameters such as total cholesterol (TC), low-density lipoprotein (LDL), high-density lipoprotein (HDL), triglycerides (TG), and fasting blood glucose (FBG) levels were obtained from the medical records.

Our research is a continuation (retrospective study) of the studies we conducted earlier on the same group of patients. The previous studies were conducted in MLWH before the implementation of cART and one year after antiretroviral therapy, and the results were published [26,27,28].

### 2.2. Institutional Review Board Statement

This study was conducted in accordance with the Declaration of Helsinki, and the protocol was approved by the Bioethical Committee of the Medical University of Wroclaw (KB-246/2023). Written informed consent to participate in the study was obtained from all participants.

### 2.3. Material for Research

The research material was plasma obtained from whole blood, which was collected from fasting subjects into test tubes with an anticoagulant (tubes with EDTA, Sarstedt, Nümbrecht, Germany). The tubes were centrifuged in an MPW-350 laboratory centrifuge (MPW Instruments, Warsaw, Poland) at 1500× *g* for 10 min to separate the plasma. The plasma was placed in microtubes (Eppendorf AG, Hamburg, Germany) and stored at −80 °C until the start of the study.

### 2.4. Test Methods

The concentrations of the tested parameters were measured using enzyme-linked immunosorbent (ELISA) methods. All tests were performed in accordance with the manufacturers’ instructions. The tests included the Human Sirtuin 1 ELISA Kit (Cat. No E2557Hu), Human Sirtuin 3 ELISA Kit (Cat. No E2559Hu), Human Sirtuin 6 ELISA Kit (Cat. No E2562Hu), Human Irisin ELISA Kit (Cat. No E3253Hu), Human Growth Differentiation Factor8 ELISA Kit (Cat. No E3058Hu), Human Peptide YY ELISA Kit (Cat. No E1369Hu), Human Glucagon-like Peptide 1 ELISA Kit (Cat. No E0022Hu), Human Dipeptidyl peptidase 4 ELISA Kit (Cat. No E6631Hu), Human Fetuin A ELISA Kit (Cat. No E1386Hu), Human Pentraxin 3 ELISA Kit (Cat. No E1938Hu), Human Stromal Cell Derived Factor 1 ELISA Kit (Cat. No E3353Hu), and Human Regulated on Activation in Normal T-cell Expressed and Secreted/C-C Motif Chemokine 5 ELISA Kit (Cat. No E3663Hu) from the Bioassay Technology Laboratory (BT Lab; Shanghai Korain Biotech Co Ltd., Shanghai, China); Human IL-4 Immunoassay (Cat. No D4050; R&D Systems, Inc., Minneapolis, MN, USA); Human IL-7 ELISA Kit (Cat. No EHIL7; Thermo Fisher Scientific Inc., Waltham, MA, USA); and Human IL-15 Immunoassay (Cat. No D1500; R&D Systems, Inc., Minneapolis, MN, USA).

The absolute number of CD4+ and CD8+ T cells was examined in whole blood collected with anticoagulant (tubes with EDTA, Sarstedt, Poland). CYTO-STAT tri-CHROME CD8-FITC (fluorescein isothiocyanate)/CD4-RD1 (phycoerythrin) reagents were used (Beckman Coulter Inc. Brea, CA, USA). The reagent was a mix of mouse monoclonal antibodies, which enables the simultaneous identification and determination of the total number of CD4+T and CD8+T lymphocytes in the blood. Samples were analyzed using a Navios EX flow cytometer (Beckman Coulter Inc., Brea, CA, USA).

HIV-1 RNA viral loads were measured using the cobas^®^ HIV-1 real-time quantitative assay (Roche Diagnostics GmbH, Mannheim, Germany) performed using the Cobas^®^ 4800. The lower detection limit was 40 HIV RNA copies/mL.

### 2.5. Statistical Analysis

The statistical analysis was performed using the Statistica 13.3 program (StatSoft, Poland). All quantitative variables were examined using the Shapiro–Wilk test to determine the type of distribution. Comparisons of quantitative variables between groups were performed using the Mann–Whitney U-test and the Friedman test with post hoc Nemenyi. In all analyses, a *p*-value < 0.05 was considered significant.

## 3. Results

### 3.1. Study Population

All demographic and clinical data of the study and control groups are presented in Table 1.

The level of biochemical parameters such as age, FBG, and TC did not differ significantly compared to the control group (*p* > 0.05). The level of HDL was significantly lower, and the level of LDL was higher in the MLWH group compared to the control group.

### 3.2. Assessment of Immunological Parameters in a Group of MLWH

The parameter results concerning the immune status of MLWH are presented in Table 2.

After analysis of the MLWH group, six people with HIV had CD4+ T lymphocytes below 500 cells/µL, and six people had CD8+T lymphocytes above 1000 cells/µL. Eighteen MLWH had ratio values above 1. Eight men had more than 40 HIV RNA copies/mL.

### 3.3. A Panel of Parameters in the Plasma of MLWH and the Control Group with Statistical Analysis

Table 3 shows the results and statistical analysis for the studied parameters in the MLWH and control groups.

It was shown that the levels of SIRT-1, SIRT-3, SIRT-6, IRS, GLP-1, DPP-4, FETU-A, PTX3, and IL-4 were higher in the MLWH group compared to levels in the control group.

However, statistically significant differences were only found for SIRT-6 (2.2-fold), IRS (1.2-fold), and IL-4 (1.2-fold). MSTN, PYY, RANTES, IL-7, and IL-15 had lower levels in the MLWH group compared to the control group, but statistically significant differences were demonstrated only for RANTES (1.6-fold) and IL-7 (1.9-fold).

### 3.4. A Panel of Demographic, Immunological, and Biochemical Data of MLWH Based on the Type of cART

Table 4 shows the demographic, immunological, and biochemical data of MLWH depending on the type of cART regimen used. One type of antiretroviral therapy combined NRTIs with an INSTI, and the other used NRTIs with a PI.

There were no significant differences in the levels of biochemical parameters and immune status parameters between the MLWH subgroup treated with an NRTI + INSTI regimen and the subgroup of patients treated with an NRTI + PI regimen.

Table 5 shows the results of the examined parameters depending on the type of cART regimen used. One type of antiretroviral therapy combined NRTIs with an INSTI, and the other used NRTIs with a PI.

Taking into account the type of antiretroviral therapy implemented, the levels of tested parameters, apart from IL-15 and IL-4, were higher in the group treated with an NRTI + INSTI regimen compared to the results obtained in the group treated with an NRTI + PI regimen. However, the differences were statistically significant only for MSTN, PTX3, RANTES, and IL-7.

### 3.5. A Panel of Parameters of MLWH Concerning theCD4+/CD8+ Ratio

Table 6 shows the results of the MLWH group concerning the CD4+/CD8+ ratio values (below and above 1).

In the MLWH group in which the CD4+/CD8+ ratio was lower than 1, median levels were not significantly higher for all parameters except IL-7 compared to the MLWH group in which the CD4+/CD8+ ratio was higher than 1.

### 3.6. A Panel of Parameters Concerning the Age of MLWH

Table 7 shows the results of parameters in the MLWH group concerning age (below and above 40 years).

Taking into account age, statistically significantly higher median levels were observed for almost all parameters except IL-4 and IL-7 in MLWH under 40 years of age. The most significant differences were demonstrated for SIRT-1 (1.4-fold) and FETU-A (2.4-fold).

### 3.7. Comparative Analysis Panel of Parameters in the MLWH Group before, after 1 Year, and after 5 Years of Antiretroviral Therapy

**Figure 1:**
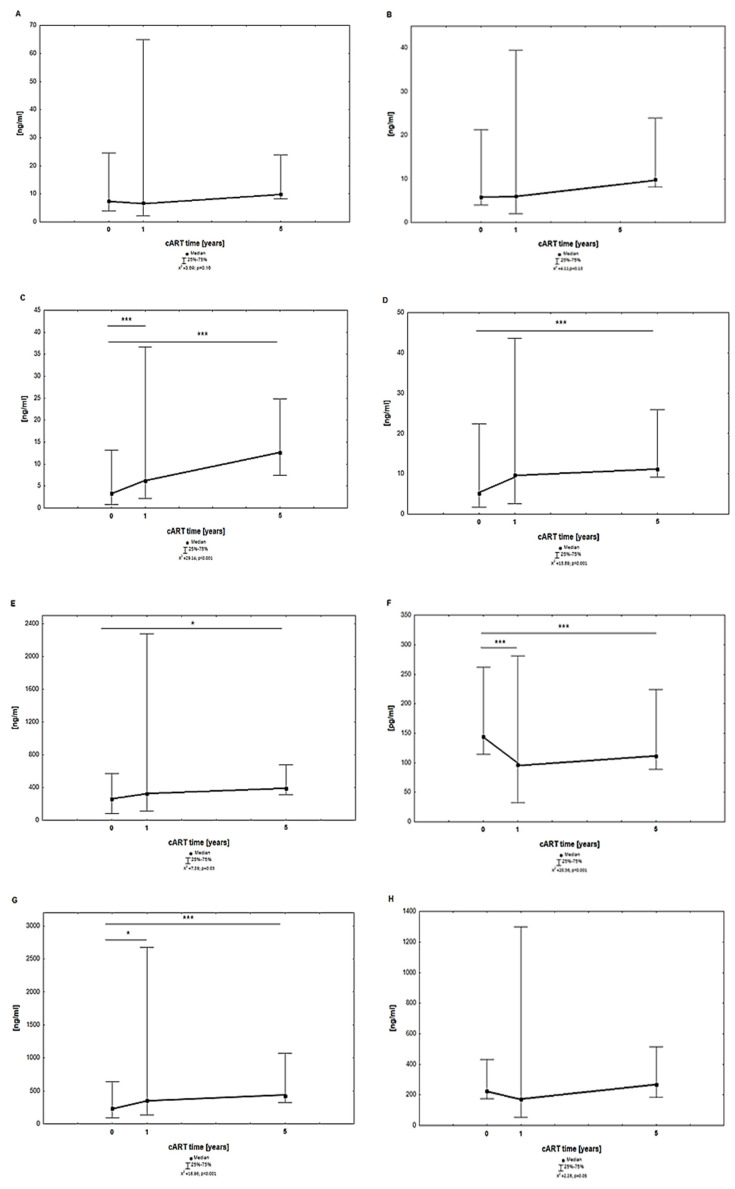
Results of SIRT-1 (**A**), SIRT-3 (**B**), SIRT-6 (**C**), IRS (**D**), MNST (**E**), PYY (**F**), GLP-1 (**G**), and DDP-4 (**H**) in the plasma of the MLWH before, 1 year after, and 5 years after cART. Abbreviations: DPP-4—dipeptidyl peptidase IV; GLP-1—glucagon-like peptide-1; IRS—irisin; MSTN—myostatin; PYY—peptide YY; SIRT, sirtuin; *** *p* < 0.001; * *p* < 0.05 statistical significance.

Figure 1 shows the results (median) of parameters characterizing metabolic disorders—SIRT-1, -3, and -6; IRS; MNST; PYY; GLP-1; and DDP-4—before, after 1 year, and after 5 years of cART use in MLWH.

The median levels of SIRT, IRS, MNST, GLP-1, and DDP-4 in MLWH increased after 5 years of cART compared to the results obtained before therapy and one year after cART, but statistically significant differences were found only for SIRT-6, IRS, MNST, and GLP-1 when comparing before with 5 years of treatment. Additionally, a statistically significant difference was observed for SIRT-6 and GLP-4 between the median level before treatment and after one year of cART. The level of median PYY decreased significantly after one year of therapy compared to the value before therapy and then increased significantly after 5 years of cART compared to the value before. For DDP-4, despite an increase in the median levels during therapy, no significant differences were found.

**Figure 2:**
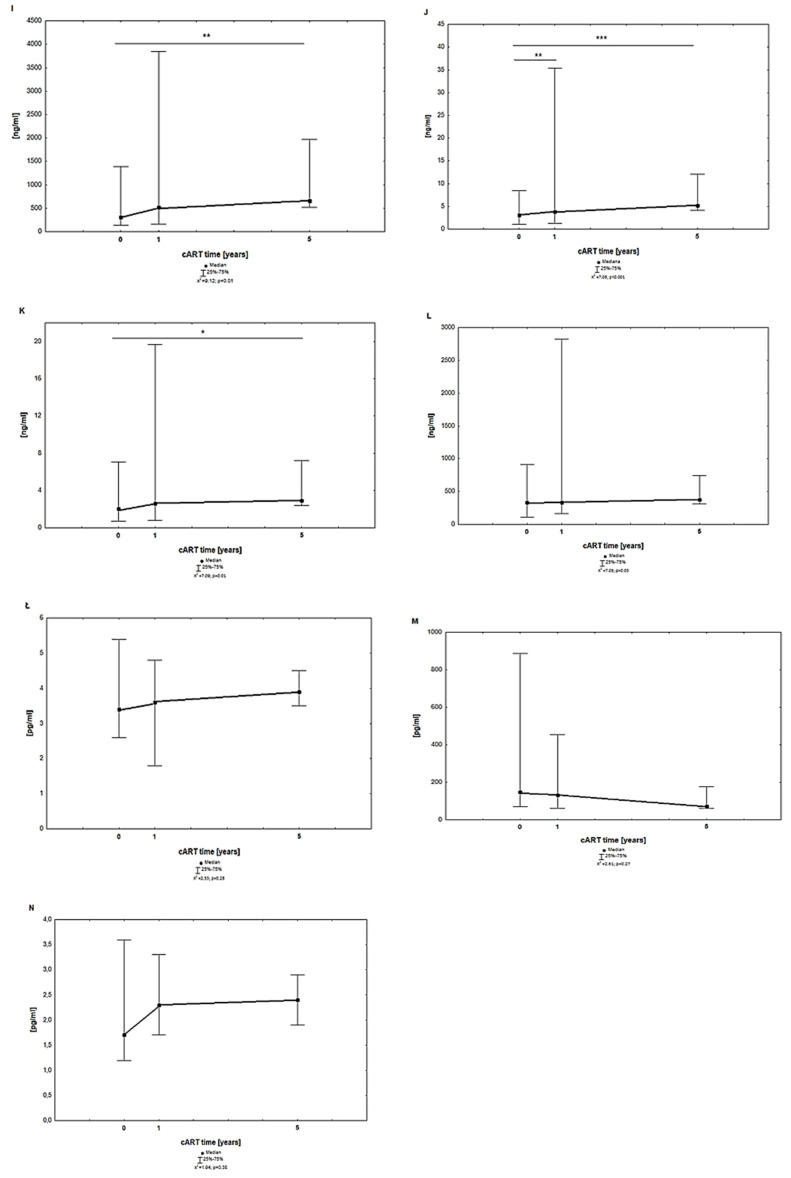
Results of FETU–A (**I**), PTX3 (**J**), SDF-1 (**K**), RANTES (**L**), IL-4(**Ł**), IL-7 (**M**), and IL-15 (**N**) in the plasma of the MLWH before, 1 year after, and 5 years after cART. Abbreviations: FETU-A—fetuin-A; IL—interleukin; PTX3—pentraxin 3, RANTES—regulated on activation, normal T cell expressed and presumably secreted; SDF-1—chemokine stromal cell-derived factor 1; *** *p* < 0.001; ** *p* < 0.01; * *p* < 0.05 statistical significance.

Figure 2 shows the median results of parameters characterizing the inflammation state and the immune system response: FETU-A, PTX3, SDF-1, chemokine RANTES, IL-4, IL-7, and IL-15.

For FETU-A, PTX3, and SDF-1, a statistically significant increase in median levels was observed after 5 years of cART compared to the results obtained prior to treatment. A significant difference between the median levels obtained before treatment and at one year after cART was detected for PTX3. The median levels before, 1 year after, and 5 years after cART were similar for RANTES, IL-4, IL-7, and IL-15 and did not differ significantly.

### 3.8. Results of Parameters in the MLWH Subgroups Treated with Two Therapeutic Regimens (INSTIs and PIs) before, 1 Year after, and 5 Years after cART

**Figure 3:**
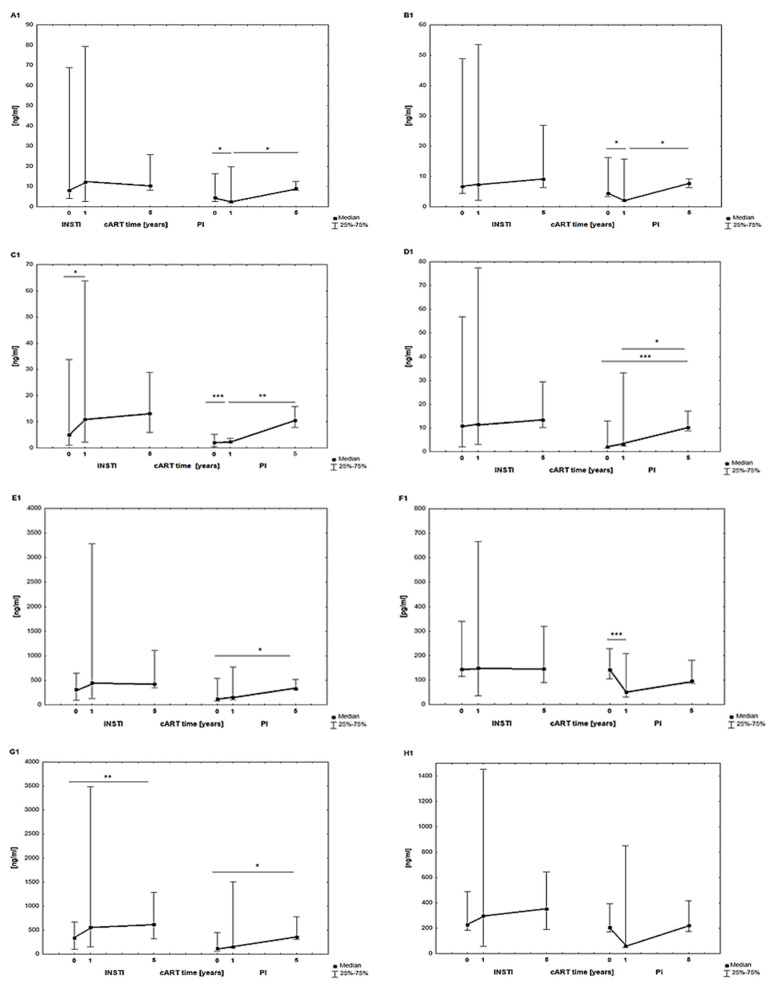
Results of SIRT-1 (**A1**), SIRT-3 (**B1**), SIRT-6 (**C1**), IRS (**D1**), MNST (**E1**), PYY (**F1**), GLP-1 (**G1**), and DDP-4 (**H1**) in MLWH subgroups treated with two therapeutic regimens (INSTIs and PIs) before, 1 year after, and 5 years after cART. Abbreviations: DPP-4—dipeptidyl peptidase IV; GLP-1—glucagon-like peptide-1; IRS—irisin; MSTN—myostatin; PYY—peptide YY; SIRT—sirtuin; *** *p* < 0.001; ** *p* < 0.01; * *p* < 0.05 statistical significance.

Figure 3 shows the results (median) of parameters characterizing metabolic disorders, including SIRT-1, -3, and -6; IRS; MNST; PYY; GLP-1; and DDP-4, before, after 1 year, and after 5 years of cART use in the MLWH subgroups treated with two therapeutic regimens (INSTIs and PIs).

Parameter levels of SIRT-1 (A1), SIRT-3 (B1), SIRT-6 (C1), IRS (D1), MNST (E1), PYY (F1), GLP-1 (G1), and DDP-4 (H1) were higher both at the time of cART initiation and after one and five years of therapy in the subgroup of MLWH treated with INSTIs compared to MLWH treated with PIs. A significant increase within one year of cART, when INSTIs were used, was noted for SIRT-6 (C1) and within five years for GLP-1 (G1).

In the subgroup of MLWH treated with PI regimens, a significant increase in the levels of SIRT-1 (A1), IRS (D1), MNST (E1), and GLP-1 (G1) was observed from the moment of cART initiation, during the first year, and over the next four years of therapy.

However, for SIRT-1 (A1), SIRT-3 (B1), and PYY (F1), after one year of cART therapy with PIs, the levels of these parameters decreased significantly (except for DPP-4 (H1), for which the decrease was not significant). Over the next four years of cART therapy with PIs, the levels of SIRT-1 (A1) and SIRT-3 (B1) increased significantly.

**Figure 4:**
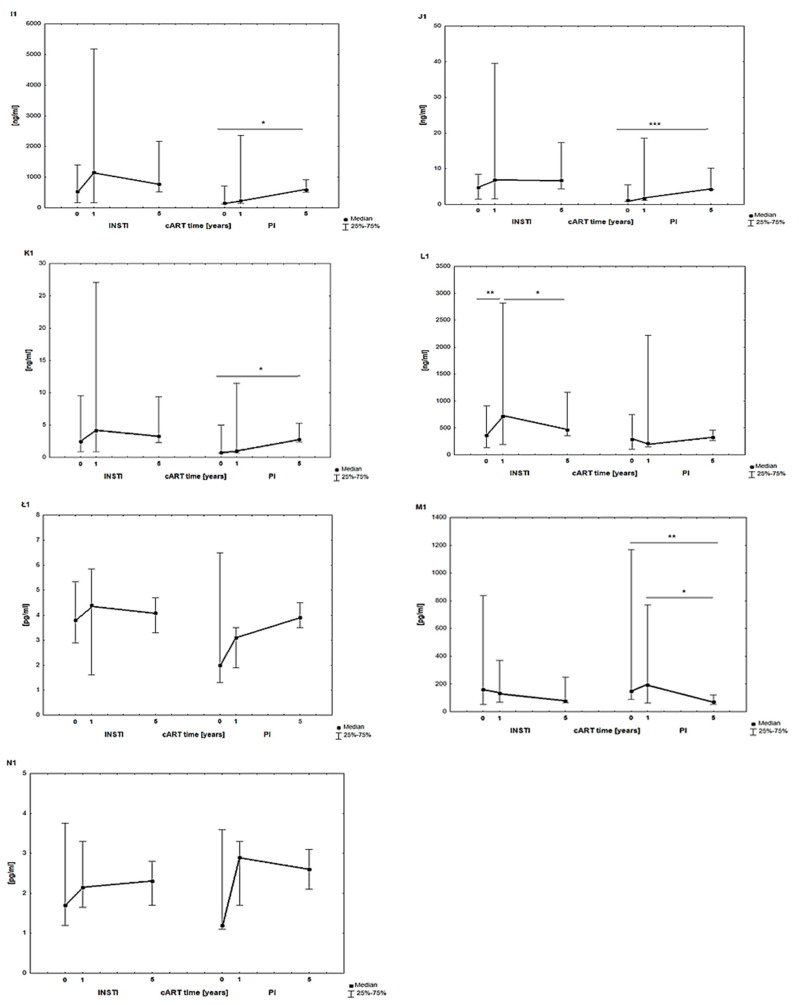
Results of FETU–A (**I1**), PTX3 (**J1**), SDF-1 (**K1**), RANTES (**L1**), IL-4 (**Ł1**), IL-7 (**M1**), and IL-15 (**N1**) in MLWH subgroups treated with two therapeutic regimens (INSTIs and PIs) before, 1 year after, and 5 years after cART. Abbreviations: FETU-A—fetuin-A; IL—interleukin; PTX3—pentraxin 3, RANTES—regulated on activation, normal T cell expressed and presumably secreted; SDF-1—chemokine stromal cell-derived factor 1; *** *p* < 0.001; ** *p* < 0.01; * *p* < 0.05 statistical significance.

Figure 4 shows the results of the medians of the parameters characterizing an inflammatory state and the immune system response: FETU-A, PTX3, SDF-1, chemokine RANTES, IL-4, IL-7, and IL-15 before, after 1 year, and after 5 years of cART use in the MLWH subgroups treated with two therapeutic regimens (INSTIs and PIs).

The levels of FETU-A (I1), PTX3 (J1), SDF-1 (K1), RANTES (L1), and IL-4 (Ł1) were higher in the subgroup of MLWH treated with INSTIs compared to PIs. An increase in the levels of these parameters was observed after one year of cART (significant for RANTES (L1)), and then there was a decrease in levels over the following four years (significant for RANTES (L1)).

The same parameters showed an increase during the five-year course of therapy with PIs, which were significant for FETU-A (I1), PTX3 (J1), and SDF-1(K1). Only IL-7 (M1) and IL-15 (N1) showed higher levels in PI-treated MLWH, which were observed after 1 year of cART. However, after five years of cART, interleukin levels were similar in both subgroups treated with INSTIs and PIs.

## 4. Discussion

Thanks to the introduction of cART, HIV infection is no longer considered a fatal disease and has gained the status of a chronic disease. Years of observation have allowed us to notice that PLWH have an increased risk of developing comorbidities, such as metabolic disorders, cardiovascular diseases, diabetes, chronic kidney disease, osteopenia, osteoporosis, etc. [2]. The risk in this population is higher than in the general population, which may be the result of multiple factors, i.e., chronic immune activation, chronic inflammation, virus activity, advancing age, and side effects of antiretroviral therapy [3,4,29]. Multi-drug regimens are recommended and widely used despite the fact that some of them may influence a greater development of metabolic disorders in some HIV-positive people, especially NRTIs and tenofovir alafenamide [5].

An analysis of 15 parameters displayed significant changes in 5 of them. We showed significantly higher median levels in MLWH compared to the control group for SIRT-6, IRS, and IL-4 and significantly lower levels for RANTES and IL-7. We observed a significant difference in the increase in median IL-4 levels in MLWH compared to HIV-uninfected people, which may be due to several years of cART.

The median levels of other parameters, although higher in MLWH, did not differ significantly from the results obtained in the control group.

SIRT-6 is mainly known as an important agent in the regulation of insulin secretion and regulator of glucose homeostasis through uptake and gluconeogenesis processes. SIRT-6 interacts with insulin-dependent glucose transporter type 4 (GLUT-4) to prevent hypoglycemia [30,31].

Some studies suggest the involvement of SIRT-6 in the pathogenesis of HIV infections. So far, there is insufficient literature regarding the potential mechanisms of action or signaling pathways of this sirtuin in HIV infections.

According to available data on IRS in HIV-infected people with metabolic syndrome, this parameter is significantly lower compared to control groups, while in PLWH without diabetes, it is significantly higher [16,32,33].

In our study, a significant increase in IRS was observed in MLWH undergoing cART with diabetes excluded compared to the control group. Subsequent years of cART therapy and the emergence of carbohydrate disorders may lead to a decrease in IRS levels in MLWH [16].

Our results, in which we noted a significant increase in IL-4 levels in MLWH compared to uninfected people, may suggest the presence of chronic inflammation associated with HIV infection and the effect of cART.

RANTES is an agonist of the CCR5 receptor, which is the target of entry inhibitors used in antiretroviral therapy and is considered a limiting factor in the progression of HIV [22,34].

This protective role played by RANTES in HIV infections could translate into a significant decrease in this parameter’s levels in MLWH, as demonstrated in our study [22,34]. Additionally, RANTES levels were significantly lower in MLWH treated with PIs compared to the INSTI-treated group.

In the study by Aukrust et al. [35], RANTES levels were significantly elevated in the serum of HIV-infected people (men and women) in all clinical stages of HIV-1 infection compared to the control group. During 16 weeks of indinavir therapy (PI), there was an increase in circulating RANTES. The decline in RANTES levels, along with disease progression, is compatible with RANTES having a beneficial role in HIV-1-infected patients.

Our study found no significant increase in RANTES levels over five years of cART. Differences in the results may be due to several reasons. Firstly, the duration of cART use (five years in our study); secondly, a different treatment regimen was implemented based on new-generation drugs (ritonavir-boosted lopinavir or cobicistat-boosted darunavir); and thirdly, we studied only HIV-infected men.

IL-7 can be considered a marker of immune system recovery during antiretroviral therapy and may be useful in monitoring treatment progress. Increased IL-7 concentrations are observed in HIV-infected people, which has also been confirmed by our research [24]. The decrease in IL-7 levels indicates proper immune reconstruction in HIV-infected people. The level of IL-7 at time 0 (before cART) was higher compared to the control (which is visible in Figure 2M) and began to decrease during therapy, which indicates an improvement in the immune status of MLWH.

In our study, it was observed that the parameters in the MLWH group were higher in men treated with NRTI + INSTI regimens compared to MLWH treated with NRTI + PI regimens. The exceptions were IL-4, the median levels of which were the same in both cART regimens, and IL-15, the median levels of which were higher in the PI regimen. For PTX3, RANTES, IL-7, and MNST, the differences were statistically significantly higher in MLWH treated with INSTI regimens.

There is only limited information in the available literature regarding MSTN in the course of HIV infections. It was shown that the concentration of MSTN in HIV-infected men undergoing cART with significant weight loss was significantly higher compared to the control group [36]. MSTN levels were positively correlated with FBG levels and the homeostatic model assessment of insulin resistance (HOMA-IR) index [37]. MSTN levels were not significantly lower in people treated with NRTI + PI regimens at one year compared to PLWH treated with NRTI + INSTI regimens [26].

In our study, the MNST level increased significantly over five years of PI therapy, while in the subset of MLWH treated with INSTIs, the MNST level increased nonsignificantly after one year and decreased over the next four years of therapy. It should be recalled that the level of MNST in cART did not differ significantly compared to controls after five years. Such changes in MNST levels (a marker associated with changes occurring in adipose and muscle tissue) were probably related to the effect of drugs on the fat/muscle mass of MLWH. According to literature data, both drug subgroups have different effects on the development and breakdown of adipose tissue in HIV-infected people [11].

In our studies, after 5 years of NRTI + INSTI use, the median MNST levels (similar to RANTES and IL-7 levels) were statistically significantly higher compared to the group of MLWH treated with NRTI + PI.

Similar to MNST, changes in PTX3 levels occurred in treatment with two cART regimens. PTX3 is released as a result of tissue damage from inflammatory reactions, is involved in modulating the activation of the complement system, and plays a key role in the reconstruction of damaged tissues thanks to its interaction with fibrin and plasminogen. It is synthesized in response to fragments of pathogens (including lipopolysaccharide) and pro-inflammatory cytokines, including TNF-α and IL-1β [19,38]. PTX3 is also considered a limiting factor in the development of bacterial, fungal, and viral infections and a prognostic indicator in the course of infection; its concentration is inversely proportional to the severity of infection [39,40]. So far, there are no data on changes in the concentration of this parameter in the course of HIV or antiretroviral therapy. Our research shows that the use of PI-based regimens has a positive effect on the increase in PTX3, i.e., the reduction in chronic inflammation.

The RANTES level was similar when using the PI-based regimen, i.e., it did not show a significant effect. However, in the subgroup of MLWH treated with INSTIs, it increased significantly after one year and reached a result comparable to that in the control group, and over the next 4 years, it decreased significantly, reaching a median value almost two times lower than that obtained in the control group.

Benyoucef et al. [41] studied the relationship between RANTES synthesis and the progression of infection in HIV-infected humans. They noted that low levels of RANTES were associated with a greater risk of disease associated with a type II immune response and a reduced type 1 immune response. RANTES synthesis was positively associated with interferon-gamma, which is crucial for type 1 immunity. Therefore, measuring RANTES levels in response to the presence of HIV may be useful in assessing the immune status and risk of increased viral replication in HIV-infected people.

The CD4+/CD8+ ratio is a marker of chronic inflammation and immunosenescence, as well as a predictor of long-term mortality in both HIV-infected and uninfected populations. In healthy people, it is 1; therefore, when antiretroviral therapy is administered to PLWH, efforts are made to maintain this value [42].

Our study did not show statistically significant differences between the levels of the tested parameters in the MLWH group being affected by the value of the CD4+/CD8+ ratio, below or above 1. It should be emphasized that over half of MLWH had a CD4+/CD8+ ratio above 1, indicating good immune recovery. It was noted that, apart from IL-15, the median parameters were higher in the MLWH group when the CD4+/CD8+ ratio was below 1. The median levels of IL-15 were higher in MLWH when the CD4+/CD8+ ratio was above 1.

IL-15 determines the cytotoxicity and proliferation of NK cells, playing a key role in host defense by directly killing virus-infected cells and in the synthesis of interferon-gamma (INF-γ), as well as the activation of macrophages. HIV-induced IL-15 gene activation is associated with massive proliferation of CD8+ T lymphocytes and the maintenance of virus-specific memory T cells. IL-15 probably has a beneficial effect on the course of HIV infection in a mechanism that depends on macrophages and stimulates them to produce chemokines such as IL-8 and MCP-1, increasing the effectiveness of the immune reaction at the site of inflammation. Decreased IL-15 expression has been demonstrated in HIV-infected patients. Moreover, IL-15 production by peripheral blood mononuclear cells was significantly reduced in antiretroviral-naïve or ineffectively treated patients [25].

In our studies, the increase in LI-15 in MLWH with a CD4+/CD8+ ratio > 1 may indicate the effectiveness of the implemented therapy and immunological reconstruction [27,43].

The median levels of the parameters (except IL-4, IL-7, and IL-15) were statistically significantly higher in the MLWH group of people under 40 years of age compared to men aged 40 and over.

Perhaps this is related to the more intense reaction of the younger body to drugs, or maybe because HIV infection is associated with faster aging of the body and the appearance of comorbidities. HIV compounds the effect of aging on the immune system. This explains why the progression of HIV in older patients is usually more pronounced. In one case-control study, it was found that both HIV-infected and noninfected older individuals had a significantly lower proportion of functional cytotoxic T cells when compared to younger patients [44].

Other studies have found that age is a predictor of HIV progression [45].

Data regarding the clinical, immunological, and virological benefits of cART in older HIV-infected patients have been conflicting. Early in the cART era, data suggested that age was inversely proportional to the rate of immune system recovery [46].

This means that the immune recovery in older patients was lower than that in younger patients. Results from the largest cohort study comparing virological and immunological responses found that the virological response was better in elderly patients; however, the immunological response was poor in this population [47].

Data on the toxicities of antiretrovirals in older patients are very limited. This is because most studies on antiretroviral metabolism exclude older patients with comorbid conditions.

The most significant difference was demonstrated for SIRT-1 and FETU-A.

The available research on SIRT-1 focuses primarily on its role in glucose metabolism, lipid metabolism, and aging. SIRT-1 is also the most widely known sirtuin in terms of immune regulation and host protection against various infections [28,48].

FETU-A is considered a multifunctional protein that is involved in, among others, metabolic disorders, insulin resistance, T2DM, and anti-inflammatory effects. During inflammation, the level of FETU-A decreases, but after infection, it increases again, which can be used to monitor the course of HIV infections [49].

The observation of high levels of SIRT-1 and FETU-A, especially in the group of younger (<40 years) MLWH, may indicate the effectiveness of cART and reduced inflammation associated with HIV [50].

A key part of our study was to compare the results of parameters determined after 5 years of cART to the results obtained before and one year after the initiation of antiretroviral therapy. The results of the parameters regarding the studied MLWH group before and one year after cART, which were used to perform a comparative analysis with the results obtained after five years of cART, were previously published by us [19,33,37].

There were significantly higher levels of SIRT-6, PYY, GLP-1, and PTX3 (for the mentioned parameters, *p* ≤ 0.001) obtained after 5 years of cART compared to the results before treatment. Also, after one year of cART, these parameters were significantly higher than the values obtained before treatment.

Of the three sirtuins tested, only SIRT-6 showed a significant increase over 5 years of cART. SIRT-6 interacts with insulin-dependent glucose transporter type 4 (GLUT-4), preventing hypoglycemia. Drugs from the PI group inhibit the action of GLUT-4 in peripheral tissues, causing impaired insulin secretion and contributing to the development of insulin resistance [7].

Literature data on GLP-1 secretion and concentration in PLWH are ambiguous. Elevated meal-stimulated GLP-1 levels have been demonstrated in HIV-infected individuals with insulin resistance compared to normoglycemic individuals [51].

Only PYY, of all the parameters tested, showed a significant decrease after 1 year and increased only slightly after 5 years of cART.

An inverse correlation has been demonstrated between PYY levels and cardiovascular risk factors (diabetes, hypertension, and hypercholesterolemia) and the risk of cardiovascular events [14]. However, there are still no literature data regarding potential changes in PYY activity or concentration in HIV-infected patients.

Of these parameters, only SIRT-6 showed a significant increase compared to controls; the remaining ones (GLP-1 and PYY), although they increased, were not higher than the values obtained in the control group. These results suggest SIRT-6 may be useful for monitoring glucose metabolism disorders in MLWH on long-term cART, regardless of the treatment regimen.

The increase in levels over 5 years of cART involved parameters characterizing metabolic disorders: IRS and MSTN, but only IRS levels were statistically significantly higher compared to the control, which was already noticed in another study on HIV-infected people [16]. The literature indicates a protective role of IRS in the development of metabolic disorders and obesity. IRS has anti-inflammatory effects, increases the sensitivity of cells to insulin, and inhibits the process of gluconeogenesis [10]. However, the receptor for IRS and its exact mechanism of action are still unknown. Research on irisin activity may also be useful in the context of comorbidities of HIV infections [9]. An increase in IRS levels in MLWH over 5 years of cART is a positive finding during HIV infections.

Among the inflammation parameters, an increase in levels during the 5 years of therapy was demonstrated for PTX3, FETU-A, and SDF-1, where the levels of the first two parameters were higher, although not significantly compared to the control group.

FETU-A is a negative acute-phase protein, and its reduced synthesis is observed in the course of inflammation. FETU-A is an inhibitor of the insulin receptor tyrosine kinase in muscle, liver, and adipose tissue, thus promoting the development of insulin resistance and T2DM [18,52]. FETU-A also inhibits the expression of adiponectin and peroxisome proliferator-activated receptor γ (PPARγ) and activates toll-like receptor 4 (TLR 4) on the surface of adipocytes and immune system cells, reducing the expression of the pro-inflammatory nuclear factor NF-kB and pro-inflammatory cytokines, which also predisposes to metabolic disorders [52]. The observed increase during cART, on the one hand, indicates a reduction in inflammation, but on the other hand, it may influence the development of metabolic dysfunctions associated with antiretroviral therapy.

The importance and exact mechanism of action of SDF-1 in HIV infections is unclear throughout the entire life cycle of the virus. SDF-1 inhibits the entry of X4-tropic viruses into the cell, probably increases the virulence of CCR5-tropic strains, and stimulates the action of the Tat protein, increasing the efficiency of viral DNA synthesis. Higher SDF-1 levels are considered a predictor of insufficient immune system recovery despite effective antiretroviral treatment. As data from the literature show, in patients whose therapy turned out to be ineffective, the SDF-1 concentration is significantly higher than in patients in whom a significant recovery and a larger number of CD4+ cells were observed [21]. However, there are still insufficient data on changes in SDF-1 concentrations during HIV infections and cART use.

Despite the increase in medians (apart from IL-7, for which the median decreased) of the remaining parameters—SIRT-1, SIRT-3, DPP-4, RANTES, IL-4, and IL-15—no significant differences were found after 5 years of cART. The levels of all tested parameters in MLWH did not show a statistically significant increase from 1 to 5 years, i.e., 4 years of cART.

Due to the increasing life expectancies of patients treated with cART and the increasing average age of a patient with a newly diagnosed retroviral infection, control of the infection, as well as proper care of comorbidities, such as metabolic diseases, cardiovascular disease, lipid metabolism disorders, osteoporosis, and chronic kidney disease, should be goals of therapy. In the face of the increasing duration of antiretroviral treatment, close monitoring of the side effects of antiretroviral drugs, in particular, the long-term effects contributing to the development and progression of chronic diseases, plays an increasingly important role [53].

That is why we attempted to examine parameters that would reflect the scale of changes in HIV-infected people undergoing cART. The panel of parameters we selected in a fragmentary way allows us to assess metabolic dysfunctions, the presence of chronic inflammation, and the degree of immune system regeneration in MLWH during antiretroviral therapy. Based on our research, we noticed some changes in the levels of tested parameters depending on cART, as well as the type of therapy and age of patients. The development of such studies will allow us to find the best panel of parameters that can be used for the early detection of various dysfunctions related to the use of cART and for proper monitoring of therapy.

When conducting this study, we wanted to select non-routine parameters related to possible metabolic disorders and inflammation in MLWH treated with several years of cART. We initially assumed that cART would affect the levels in the patient’s plasma. The analysis of the studies confirmed changes in the levels of several parameters in the MLWH plasma (especially SIRT-6, IRS, MSTN, GLP-1, FETU-A, PTX3, and RANTES) associated with the negative or positive effect of cART. This convinced us of the validity of continuing our research and searching for other parameters helpful in the assessment and monitoring of antiretroviral drug regimens used in MLWH.

To sum up, we observed higher levels of parameters (except IL-15) in the MLWH subgroup treated with INSTIs (period of five years) compared to patients treated with PIs, among which MSTN, PTX3, RANTES, and IL-7 showed a significant difference in levels. The obtained results were more clearly demonstrated by the analysis of parameters in the subgroups treated by the two regimens, covering the time from the initiation of cART after one year and five years of therapy. We did not find similar data reported by other scientists. Moreover, younger MLWH (<40 years of age) had significantly higher levels of tested parameters (except for IL-4 and IL-7) than older patients (≥40 years of age). These data are novel; the subgroup of MLWH treated with INSTIs consisted of younger men, which would explain these results. The practical aspect of our observations is that the decision to choose a cART regimen may be based on the examination of certain new parameters, such as SIRT-6, IRS, MSTN, GLP-1, FETU-A, PTX3, and RANTES, for which the outcomes may predict the development of metabolic disorders after years of use of particular cART regimens.

### Limitations

Limitations of the study include the low number of patients per group and that we did not analyze tenofovir alafenamide (TAF, belonging to the NRTI group) for metabolic disorders comparing two groups of HIV-positive patients because the backbone in both groups usually included TAF.

## 5. Conclusions

The obtained results indicate that during HIV infections and 5 years of cART, certain changes in the levels of tested parameters reflect disorders of various natures and severities. However, it should be emphasized that the health conditions of the HIV-infected men were good, which proves the proper implementation and monitoring of cART. The lack of significant metabolic disorders or changes in the severity of inflammatory markers related to the remodeling of the immune system in MLWH is most likely due to the relatively young age of the study participants and the use of new-generation antiretroviral drugs. Continuation of this research topic seems to be justified, and deepening the knowledge about the presented parameters will allow the selection of a panel of parameters that will prove useful in predicting the development of long-term disorders related to the course of the disease.

## Figures and Tables

**Table 1:** Demographic and biochemical data of MLWH and control group with statistical analysis. Data are expressed as median and interquartile range.

Group Characteristic	MLWH (n = 54)	Control Group (n = 51)	*p*
	Me (IQR)		
Age [years]	37.50 (34.00–46.00)	39.98 (36.00–45.00)	0.39
FBG [mg/dL]	90.50 (85.00–103.00)	93.20 (88.00–99.00)	0.78
TG [mg/dL]	116.50 (86.00–158.00)	105.00 (98.00–115.00)	0.21
TC [mg/dL]	182.50 (167.00–214.50)	179.00 (165.00–195.00)	0.34
LDL [mg/dL]	121.00(106.00–132.50)	99.00 (97.00–115.00)	0.002
HDL [mg/dL]	46.00 (42.00–53.00)	74.00 (59.00–88.00)	0.03

Abbreviations: FBG—fasting blood glucose; HDL—high-density lipoprotein; IQR—interquartile range; LDL—low-density lipoprotein; MLWH—men living with HIV; Me—median; n—number of participants; TC—total cholesterol; TG—triglycerides; *p* < 0.05 statistical significance (Mann–Whitney test).

**Table 2:** Results of immunological parameters in a group of MLWH.

	Me (IQR)
CD4+ T lymphocytes [cells/µL]	647.00 (500.00–754.00)
CD8+ T lymphocytes [cells/µL]	661.00 (498.00–823.00)
CD4+/CD8+ ratio	0.93 (0.74–1.32)
HIV RNA [copies/mL]	54.00 (47.00–31,800.00)

Abbreviations: HIV RNA—HIV viral load; IQR—interquartile range; Me—median

**Table 3:** Results of parameters in the plasma of MLWH and the control group with statistical analysis.

Parameters	MLWH (n = 54)	Control Group (n = 51)	*p*
	Me (IQR)		
SIRT-1 [ng/mL]	9.90 (8.20–23.90)	8.60 (4.90–31.40)	0.36
SIRT-3 [ng/mL]	7.85 (6.40–20.70)	6.40 (4.20–27.10)	0.14
SIRT-6 [ng/mL]	12.60 (7.50–24.50)	5.70 (3.80–32.60)	0.02
IRS [ng/mL]	11.10 (9.20–25.90)	9.30 (3.40–31.50)	0.02
MSTN [ng/mL]	387.00 (312.80–678.80)	393.0 (240.20–1755.20)	0.95
PYY [pg/mL]	111.55 (89.30–224.10)	114.30 (80.30–418.10)	0.71
GLP-1 [ng/mL]	418.99 (324.87–1067.91)	360.40 (195.39–1131.00)	0.09
DPP-4 [ng/mL]	268.05 (185.20–514.80)	262.80 (124.40–780.40)	0.42
FETU-A [ng/mL]	660.50 (520.30–1965.10)	593.20 (328.70–3333.10)	0.82
PTX3 [ng/mL]	5.20 (4.10–12.10)	4.30 (2.50–20.60)	0.30
SDF-1 [ng/mL]	2.90 (2.40–7.20)	2.90 (1.80–12.20)	0.86
RANTES [ng/mL]	372.10 (311.10–745.70)	601.50 (363.40–1665.20)	0.02
IL-4 [pg/mL]	3.90 (3.50–4.50)	3.35 (2.20–4.10)	≤0.001
IL-7 [pg/mL]	74.00 (62.00–176.00)	138.85 (68.00–497.00)	0.03
IL-15 [pg/mL]	2.40 (1.19–2.90)	2.50 (1.80–3.00)	0.20

Abbreviations: IQR—interquartile range; DPP-4—dipeptidyl peptidase IV; FETU-A—fetuin-A; GLP-1—glucagon-like peptide-1; IRS—irisin; IL—interleukin; Me—median; MLWH—men living with HIV; MSTN—myostatin; n—number of participants; PTX3—pentraxin 3; PYY—peptide YY; RANTES—regulated on activation, normal T cell expressed and presumably secreted; SDF-1—chemokine stromal cell-derived factor 1; *p* < 0.05 statistical significance (Mann–Whitney test).

**Table 4:** The demographic, immunological, and biochemical data of MLWH depending on the type of cART regimen used.

Parameters	NRTI + INSTI (n = 31)	NRTI + PI (n = 23)	*p*
	Me (IQR)		
Age [years]	37.00 (32.00–42.00)	39.00 (34.00–46.00)	0.16
CD4+ T lymphocytes [cells/µL]	694.00 (539.00–842.00)	618.50 (42.50–727.00)	0.26
CD8+ T lymphocytes [cells/µL]	661.00 (446.00–822.00)	699.00 (514.00–974.00)	0.52
CD4+/CD8+ ratio	1.00 (0.83–1.32)	0.84 (0.58–1.31)	0.19
HIV RNA [copies/mL]	63.50 (40.00–76.00)	52.00 (47–31800)	0.41
FBG [mg/dL]	89.50 (83.00–104.00)	92.00 (86.00–103.00)	0.85
TG [mg/dL]	116.00 (68.00–151.00)	116.50 (94.00–222.00)	0.36
TC [mg/dL]	176.50 (167.00–197.00)	198.50 (167.00–222.00)	0.28
LDL [mg/dL]	113.00 (107.00–128.00)	125.5 (81.00–156.00)	0.37
HDL [mg/dL]	46.00 (44.00–55.00)	47.80 (40.00–52.00)	0,66

Abbreviations: see Table 1 and Table 2.

**Table 5:** Parameter results in the MLWH group depending on the type of cART.

Parameters	NRTI + INSTI (n = 31)	NRTI + PI (n = 23)	*p*
	Me (IQR)		
SIRT-1 [ng/mL]	10.40 (8.20–25.90)	9.20 (8.20–12.60)	0.26
SIRT-3 [ng/mL]	9.10 (6.40–26.90)	7.80 (6.40–9.30)	0.28
SIRT-6 [ng/mL]	13.20 (5.90–28.80)	10.50 (7.80–15.80)	0.62
IRS [ng/mL]	13.30 (10.10–29.50)	10.20 (8.80–17.10)	0.07
MSTN [ng/mL]	425.00 (350.20–1110.10)	334.40 (304.40–515.10)	0.02
PYY [pg/mL]	145.60 (90.10–319.60)	96.30 (86.20–180.90)	0.12
GLP-1 [ng/mL]	618.08 (324.87–1286.15)	354.86 (308.21–779.69)	0.07
DPP-4 [ng/mL]	351.10 (191.30–645.20)	221.10 (174.60–416.60)	0.08
FETU-A [ng/mL]	778.10 (522.80–2172.10)	587.10 (508.10–918.20)	0.12
PTX3 [ng/mL]	6.80 (4.30–17.30)	4.20 (4.10–10.20)	0.04
SDF-1 [ng/mL]	3.30 (2.30–9.40)	2.80 (2.40–5.30)	0.59
RANTES [ng/mL]	464.10 (358.80–1162.10)	328.20 (270.40–458.10)	0.01
IL-4 [pg/mL]	4.10 (3.30–4.70)	3.90 (3.50–4.50)	0.93
IL-7 [pg/mL]	76.00 (64.00–250.00)	70.00 (52.00–122.00)	0.03
IL-15 [pg/mL]	2.30 (1.70–2.80)	2.60 (2.10–3.10)	0.06

Abbreviations: see Table 3.

**Table 6:** Results of parameters in the MLWH group depending on the CD4+/CD8+ ratio value.

Parameters	CD4+/CD8+ ratio < 1 (n = 18)	CD4+/CD8+ ratio ≥ 1 (n = 36)	*p*
	Me (IQR)		
SIRT-1 [ng/mL]	10.70 (8.30–16.60)	9.15 (7.90–10.50)	0.17
SIRT-3 [ng/mL]	8.40 (7.00–17.20)	7.85 (6.30–13.50)	0.43
SIRT-6 [ng/mL]	13.10 (8.20–26.40)	9.95 (5.30–17.20)	0.58
IRS [ng/mL]	12.10 (10.10–25.40)	9.85 (8.60–16.80)	0.09
MSTN [ng/mL]	390.30 (316.40–519.50)	343.45 (304.40–587.10)	0.41
PYY [pg/mL]	117.90 (89.80–202.40)	95.25 (84.20- 177.10)	0.32
GLP-1 [ng/mL]	411.50 (329.87–1007.93)	376.52 (308.21–897.97)	0.46
DPP-4 [ng/mL]	274.50 (183.70–474.80)	223.50 (169.10–384.70)	0.45
FETU-A [ng/mL]	687.50 (572.80–1666.20)	540.05 (513.50–1479.10)	0.31
PTX3 [ng/mL]	5.10 (4.20–10.80)	4.40 (3.50–8.90)	0.29
SDF-1 [ng/mL]	3.10 (2.50–6.70)	2.55 (2.20–4.20)	0.19
RANTES [ng/mL]	410.20 (340.60–718.10)	336.45 (286.40–566.40)	0.17
IL-4 [pg/mL]	4.10 (3.70–4.70)	3.70 (3.50–4.10)	0.21
IL-7 [pg/mL]	72.00 (58.00–170.00)	76.00 (64.00–138.00)	0.60
IL-15 [pg/mL]	2.50 (2.10–2.90)	2.20 (1.90–2.60)	0.19

Abbreviations: see Table 3 and Table 4.

**Table 7:** Results of parameters in the MLWH group concerning age (below and above 40 years).

Parameters	Age < 40 Years 40 (n = 30)	Age ≥ 40 Years (n = 24)	*p*
	Me (IQR)		
SIRT-1 [ng/mL]	11.85 (9.60–28.50)	8.25 (7.50–10.45)	≤0.001
SIRT-3 [ng/mL	11.25 (7.60–29.30)	6.85 (6.15–8.40)	0.002
SIRT-6 [ng/mL]	15.65 (8.70–43.90)	8.55 (5.35–11.95)	0.003
IRS [ng/mL]	16.50 (10.30–50.10)	9.95 (8.50–12.90)	0.003
MSTN [ng/mL]	464.30 (329.00–1167.10)	347.20 (264.55–445.30)	0.02
PYY [pg/mL]	179.00 (95.90–345.20)	91.60 (80.90–136.35)	0.002
GLP-1 [ng/mL]	818.01 (354.86–1627.68)	351.53 (293.22–603.09)	0.01
DPP-4 [ng/mL]	407.80 (214.20–741.80)	218.10 (170.60–313.70)	0.01
FETU-A [ng/mL]	1349.1(591.30–3563.10)	561.05 (471.85–713.10)	≤0.001
PTX3 [ng/mL]	8.25 (4.30–20.10)	4.20 (3.50–6.15)	0.002
SDF-1 [ng/mL]	4.65 (2.60–16.30)	2.55 (2.20–3.20)	0.002
RANTES [ng/mL]	515.25 (388.80–1531.10)	347.20 (279.95- 453.60)	0.003
IL-4 [pg/mL]	3.9 (3.70–4.70)	3.90 (3.50–4.40)	0.93
IL-7 [pg/mL]	76.00 (62.00–248.00)	73.00 (63.00–129.00)	0.52
IL-15 [pg/mL]	2.15 (1.90–2.50)	2.65 (2.25–3.35)	0.02

Abbreviations: see Table 3, Table 4 and Table 5.

## Data Availability

All data generated or analyzed during this study are included in the manuscript. Further inquiries should be directed to the corresponding author.
